# Acute Exercise Promptly Normalizes Myocardial Myosin Heavy-Chain Isoform mRNA Composition in Diabetic Rats: Implications for Diabetic Cardiomyopathy

**DOI:** 10.3390/medicina59122193

**Published:** 2023-12-18

**Authors:** Ramzi Ahmad Al-Horani, Saja Janaydeh, Bahaa Al-Trad, Mukhallad Mohammed Aljanabi, Riyadh Muhaidat

**Affiliations:** 1 Exercise Science Department, Yarmouk University, Irbid 21163, Jordan; 2 Department of Biological Sciences, Yarmouk University, Irbid 21163, Jordan; sajawaleed91@yahoo.com (S.J.); bahaa.tr@yu.edu.jo (B.A.-T.); muhaidat@yu.edu.jo (R.M.); 3 Department of Physiology, Faculty of Medicine, Jordan University of Science and Technology, Irbid 22110, Jordan; mukmoh@just.edu.jo

**Keywords:** myosin isozymes, thyroid receptors, myocardium, sedentary lifestyle, exercise training

## Abstract

*Background and Objectives:* The acute effects of exercise on the myosin heavy-chain (MHC) isoform mRNA expression and the upstream transcription factors in diabetic and non-diabetic hearts remain unexplored. We aimed to determine the acute effect of a single exercise session on the expression of left ventricular MHC, MHC-α and MHC-β, and thyroid receptor (TR), TR-α1 and TR-β, isoform mRNA in diabetic and non-diabetic rats. *Materials and Methods:* Sprague-Dawley rats were assigned to four groups: non-diabetic control (CS), diabetic exercise (DIEX), sedentary diabetic (DIS), and non-diabetic exercise (CEX). Diabetes was induced via streptozotocin injection (55 mg/kg). DIEX and CEX rats performed an exercise session (60 min at 50 m/min and 0% grade) 6–7 weeks after diabetes induction. *Results:* MHC-α mRNA was lower in DIS (*p* = 0.03) and not different in DIEX (*p* = 0.1) relative to CS. DIS showed higher MHC-β mRNA than the non-diabetic rats, CS and CEX (*p* = 0.02 and *p* = 0.009, respectively). MHC-β mRNA in DIEX was normalized to non-diabetic levels in CS (*p* = 0.3). TR-α1 was higher in DIS and not different in DIEX relative to CS and CEX (*p* = 0.03 and *p* = 1.0, respectively). In CEX, exercise did not change MHC-α, MHC-β, and TR-α1 relative to CS (*p* = 1.0). TR-β was not different between groups. *Conclusion:* In conclusion, exercise appears to acutely normalize the myocardial MHC and TR isoform mRNA expression only in the diabetic heart. These responses may induce therapeutic mechanisms other than changing the MHC isoform composition.

## 1. Introduction

Diabetic cardiomyopathy is characterized by abnormal myocardial structure and ventricular dysfunction, independent of other risk factors such as hypertension and coronary heart disease, in individuals with diabetes mellitus [1]. Individuals with diabetes experience an elevated risk of morbidity and mortality, primarily from cardiovascular complications, including heart failure [2,3,4]. The factors associated with this increased incidence of cardiovascular complications and mortality with diabetes remain incompletely identified [3]. 

Mammalian cardiac myocytes express two isoforms of myosin heavy-chain (MHC): MHC-α and MHC-β. MHC-α exhibits threefold higher calcium- and actin-activated ATPase activity compared to the MHC-β isoform [5]. The relative expression of each isoform has been shown to critically influence the contractile performance of the myocardium, where increased MHC-α expression improves while increased MHC-β expression impairs cardiac contractility [6,7]. Diabetic cardiomyopathy is characterized by a large transformation in the structural MHC composition towards the MHC-β isoform. For example, the relative proportion of myocardial MHC-β was increased from 18% to 97% 6 weeks post-diabetes induction in rats [8]. Mechanical defects in the heart contraction such as systolic dysfunction associated with diabetic cardiomyopathy have been attributed, at least in part, to shifts in cardiac MHC isoforms towards MHC-β with reduced calcium-activated ATPase activity [2,9]. In this context, various pharmacological interventions such as Dimethylurea and insulin have shown potential in improving cardiac contractile function of the diabetic heart in conjunction with a reversal of MHC isoform composition toward MHC-α predominance [9,10]. 

Exercise training is a critical, non-pharmacological component in the prevention and treatment of diabetes. It has consistently shown an ability to improve or restore the deteriorated cardiac function and contractility in both diabetic human and rodent models with established diabetic cardiomyopathy [11,12]. These exercise training-related improvements in contractility of the diabetic heart were postulated to be due to normalization of cardiac MHC isoform distribution towards MHC-α [13]. However, MHC isoform composition was not normalized in the diabetic and normal heart after long-term exercise training, despite improved cardiac contractility [13,14,15]. Consequently, it was suggested that improvements in contractile function of the diabetic heart after exercise training were induced by mechanisms other than normalization of cardiac myosin isoenzyme distribution [13]. Nonetheless, there might be a transient change in the MHC isoform mRNA expression after each exercise that may trigger other mechanisms responsible for modulating cardiac function and contractility. For example, it has been shown that MHC-α stimulates the encoding of microRNA-208, which is closely associated with heart health and disease [16,17]. The acute responses of MHC isoform mRNA expression to a single session of exercise remain poorly investigated in both the normal and diabetic heart. 

Thyroid hormone influences cardiac contractility primarily through its active metabolite triiodo-thyronine (T3) interacting with the cardiac thyroid receptors (TR), TR-α1, TR-β [18]. The activation of these receptors regulates the transcription of MHC-α and MHC-β mRNA [19]. Studies have shown that T3 administration results in an immediate upregulation of MHC-α mRNA transcription within the first 30 min. Therefore, since T3 and exercise induce similar acute cardiac responses [20], it is possible that a single session of exercise could potentially alter the expression of these interconnected genes. 

Therefore, we aimed to determine whether a single exercise session could induce acute changes in MHC isoform mRNA expression in both diabetic and non-diabetic hearts. Additionally, we aimed to assess the acute expression of TR isoform mRNA following a single exercise session, given their established role in regulating MHC isoform expression. 

## 2. Methodology

### 2.1. Animals and Experimental Design

All experimental procedures were conducted in compliance with the Guide for the Care and Use of Laboratory Animals and were reviewed and approved by the local committee at Yarmouk University (IACUC/2020/2; Approval Date: 12 February 2020). Initially, 43 male Sprague-Dawley rats were obtained from the animal house of the Department of Biological Sciences at Yarmouk University. Unfortunately, four rats from the diabetic group and one from the non-diabetic group did not survive, and three did not develop diabetes after a second Streptozotocin (STZ) dose of diabetes. Consequently, only 35 rats were included in the analysis and assigned to the following groups. Rats were assigned to one of four groups: (1) non-diabetic control (CS; n 8); (2) diabetic exercise (DIEX; n 9); (3) sedentary diabetic (DIS; n 9); and (4) non-diabetic exercise (CEX; n 9) groups. Additional rats were deliberately included in the diabetic groups in anticipation of higher likelihood of mortality and failure of diabetes induction. Rats were 10–12 weeks old and 200–250 g. Rats were housed 3–4 per cage on a 12:12 h light–dark cycle at 23–24 °C with free access to standard rodent food and water ad libitum.

STZ injection was used to induce diabetes in DIEX and DIS. Diabetes induction using STZ was conducted as previously described [21,22]. Animals received a single intraperitoneal STZ (Sigma-Aldrich, St. Louis, MO, USA) injection (55 mg/kg) after an overnight fast. For the next 24 h, rats were provided with 10% sucrose water ad libitum to avoid hypoglycemia. The rats were then fed with normal chow and provided with free access to water ad libitum. Forty-eight hours after the STZ injection, fasting blood glucose was measured using a One-touch blood glucose monitoring system to test for diabetes induction. Diabetes was confirmed if fasting blood glucose was >250 mg/dL. Rats who did not develop hyperglycemia were injected with a second dose of STZ and followed similar criteria to confirm diabetes. The animals (3 rats) that did not develop diabetes after the second STZ injection were excluded and not assigned to any group. Diabetic rats were left in their cages for 6–7 weeks. Previous findings have shown that cardiomyopathy develops in Sprague-Dawley rats within 4–8 weeks following STZ injection (55 mg/kg), indicated by left ventricular remodeling and diastolic dysfunction [23,24,25]. In our study, we followed similar methodological procedures as outlined in these studies, using similar rat strain, STZ dosage, and an experimental total duration of 7–8 weeks following diabetes induction. Given these similarities with the established timelines for cardiomyopathy development, we highly posit that our rats had developed cardiomyopathy by the conclusion of the experiment. 

### 2.2. Exercise Protocol 

Exercise groups DIEX and CEX were habituated to running on the treadmill for five consecutive days, starting at 28 m.min^−1^ for 10 min at 0% grade. During habituation, the speed was occasionally increased up to 50 m.min^−1^ to familiarize the rats with the subsequent main acute exercise speed. DIEX and CEX rats were completely rested for 72 h after the last habituation session to eliminate any possible residual acute effect of exercise. Thereafter, DIEX and CEX rats ran for 60 min at 50 m/min and 0% grade. Mild electrical shocks were sparingly administered when necessary to motivate the rats to continue running. DIS and CEX were kept rested in cages without any structured exercise. 

### 2.3. Tissue Removal and Preparation 

DIEX and CEX rats were decapitated at the C3–C4 level 1.5–2 h after exercise cessation. Hearts were quickly removed, rinsed with phosphate-buffered saline (PBS 1x), weighed, and dissected to separate the right and left ventricles. Left ventricle samples were immediately frozen in liquid nitrogen and stored at −80 °C. The CS and DIS animals were sacrificed and their left ventricle samples obtained and stored at −80 °C according to the same procedure. All animals in the four groups spent similar period after STZ injection. 

### 2.4. RNA Isolation and Quantification

Total RNA was isolated from left ventricular tissue according to the RNA isolation kit procedures (Direct-Zol^TM^ RNA MiniPrep, CA, USA). About 25 mg of ventricular tissue was homogenized in 600 µL of trizole reagent in bead mill homogenizer (Bead Ruptor 4, OMNI, US). In addition, DNAase treatment step and 25 μL elution were utilized. The RNA integrity was evaluated using 1.5% denaturing agarose gel electrophoresis at 60 V for 60 min. The 28S and 18S ribosomal RNA bands were undegraded at an approximate 2:1 ratio [26]. The extracted RNA was stored at −80 °C. 

RNA quantity and purity were determined using NanoDrop spectrophotometer technology (NanoDrop™ 2000, Thermo Scientific, Waltham, MA, USA). Complementary DNA (cDNA) was synthesized by reverse transcription from 500 ng of Total RNA using oligo-(dT) primer according to the manufacturer’s instructions (Cat. #RR036A, Takara, Japan). Briefly, a total volume of 10 μL reaction was prepared from the master mix, RNA template, and RNase free water. The reaction was run for one cycle of 37 °C for 15 min, 85 °C for 5 s, and indefinite time at 4 °C. 

Quantitative real-time PCR (RT-PCR) was performed in Line-Gene 9600 Real-Time PCR system (Bioer Technology, Bingjiang, China). SYBER Green Premix Ex TaqII^TM^ master mix (Cat. #RR820L, Takara, Japan) was used. The total reaction volume was 10 μL composed of SYBR green (0.4 μM), forward and reverse primers, 1:10 diluted cDNA, and nuclease-free water. Thermal cycling was performed for 40 cycles with a three-step protocol as follows: denaturation for 30 s at 95 °C, annealing for 30 s at 60 °C, and extension for 5 sec at 72 °C. The mRNA expression of MHC-α, MHC-β, TR-α1, TR-β and the control housekeeping gene Glyceraldehyde-3-Phosphate Dehydrogenase (GAPDH) were quantified (Table 1). All primers were purchased from Integrated DNA Technologies. Data from RT-PCR were analyzed using the 2^−∆∆CT^ method [27]. The change in mRNA expression was calculated by normalizing the threshold cycle (C_T_) of the target gene to C_T_ of the reference gene GAPDH. The untreated CS was used as the calibrator.

### 2.5. Statistical Analysis

Statistical analysis was performed using the Statistical Package for Social Sciences software (SPSS, Chicago, IL, USA, version 20.0). Data are presented as mean ± SD. The main effect of group on gene expressions was determined using one-way ANOVA, followed by pairwise comparisons with Bonferroni adjustments. Changes in body weight were analyzed using two-way repeated measures ANOVA with Bonferroni adjustment. Significance was set at *p* < 0.05. 

## 3. Results

### 3.1. Animals Characteristics

Table 2 represents the body weight changes (BW), heart weight (HW), and heart-to-body-weight ratio (HW/BW) during the experiment period. There was a time (*p* = 0.03) and time x group effect (*p* = 0.005) on body weight changes. The initial body weight did not differ among the groups (*p* = 0.1–1). Body weight significantly increased from baseline only in CEX (*p* = 0.001) and tended to increase in CS rats. The final body weight of DIS and DIEX mice was significantly lower than that of the non-diabetic rats (DIS vs. CS and CEX, *p* < 0.001–0.003; DIEX vs. CEX *p* = 0.02). The HW/BW ratio was higher in DIEX compared to all three groups (*p* < 0.005). There was a significant difference between groups in terms of the HW and HW/BW ratio (*p* < 0.001 and *p* = 0.03, respectively). The HW of DIS rats was lower than that of all other groups (*p* < 0.001). On the other hand, HW/BW was greater in DIEX than in all other groups (*p* = 0.004–0.04). No differences were found between the CS, CEX, and DIS rats. 

### 3.2. Gene Expression Changes 

#### 3.2.1. MHC-α mRNA

**Figure 1:**
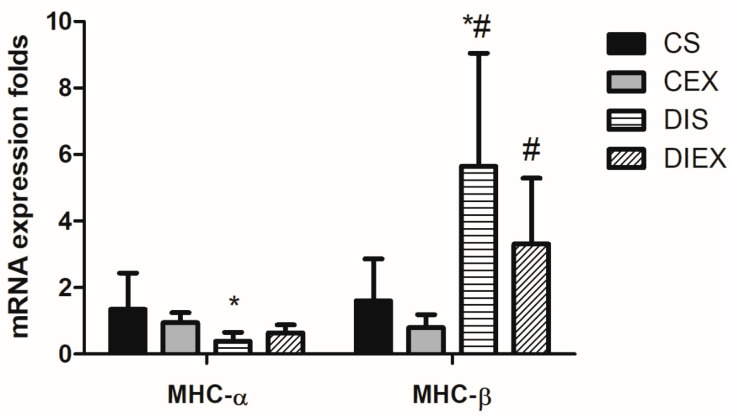
Fold change in MHC-α and MHC-β mRNA relative expression in the control sedentary (CS; n = 7), control exercise (CEX; n = 7), diabetic sedentary (DIS; n = 7), and diabetic exercise (DIEX; n = 9) mice. One-way ANOVA followed by multiple comparisons with Bonferroni adjustment was used in analysis. * Significantly different from CS at *p* < 0.05. # Significantly different from CEX at *p* < 0.05.

The analysis of variance showed a significant main effect of group on the relative expression of MHC-α mRNA (*p* = 0.02). DIS showed lower MHC-α mRNA relative expression compared to CS (*p* = 0.03). However, when diabetic rats were exposed to exercise (DIEX), MHC-α mRNA relative expression was not different from CS (*p* = 0.1). In contrast, acute exercise in the non-diabetic state (CEX) showed no effect on the expression of MHC-α relative to CS (*p* = 1.0) (Figure 1).

#### 3.2.2. MHC-β mRNA

The group had a significant effect on the relative expression of MHC-β mRNA (*p* = 0.001). MHC-β mRNA expression was significantly greater in DIS compared to the non-diabetic rats (CS and CEX) (*p* = 0.02 and 0.009, respectively). However, upon exposure to acute exercise, MHC-β mRNA expression in DIEX was not different from that in the CS (*p* = 0.1) but remained greater than in the CEX group (*p* = 0.02). There was no acute effect of exercise on MHC-β mRNA expression in in the non-diabetic state (CEX) relative to CS (*p* = 0.2) (Figure 1).

#### 3.2.3. TR-α1 mRNA

**Figure 2:**
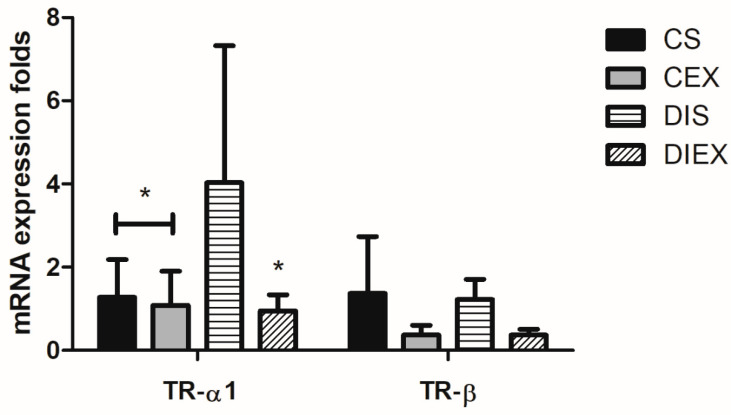
Fold change in TR-α1 and TR-β mRNA relative expression in the control sedentary (CS; n = 7), control exercise (CEX; n = 7), diabetic sedentary (DIS; n = 7), and diabetic exercise (DIEX; n = 9) mice. One-way ANOVA followed by multiple comparisons with Bonferroni adjustment was used in analysis. * Significantly different from DIS at *p* < 0.05.

The group had a significant effect on the relative expression of TR-α1 mRNA (*p* = 0.005). TR-α1 mRNA relative expression was significantly higher in DIS compared to the non-diabetic rats CS and CEX (*p* = 0.03 and 0.02, respectively). TR-α1 mRNA expression after acute exercise in DIEX was not different from the CS and CEX (*p* =1.0). Exercise did not impact TR-α1 mRNA expression in the non-diabetic state (CEX) relative to CS (*p* = 1.0) (Figure 2).

#### 3.2.4. TR-β mRNA

The group had a significant effect on the TR-β mRNA relative expression (*p* = 0.01). This was just an omnibus effect. The multiple-comparisons analysis revealed no differences between any two groups (*p* = 0.6–1.0) (Figure 2). 

## 4. Discussion

This study primarily investigated whether a single exercise session would acutely and differentially change the expression of MHC isoform mRNA in the diabetic and non-diabetic myocardium. The main findings were that sedentary diabetic myocardium expressed lower levels of MHC-α mRNA and higher levels of MHC-β mRNA compared to non-diabetic myocardium. Remarkably, exercise acutely normalized their expression to levels similar to that of normal myocardium. Conversely, acute exercise did not impact the MHC isoform mRNA expression in the normal myocardium. In addition, we explored the acute expression of thyroid receptors, TR-α1 and TR-β, in relation with myocardial MHC isoform composition. TR-α1 mRNA was elevated in the sedentary diabetic heart and was normalized to normal levels by acute exercise, whereas TR-β mRNA remained unchanged across all conditions. 

Exercise training has been shown to improve contractile function of the diabetic heart [14]. These improvements might be linked to the cumulative effects of consecutive acute molecular responses. Previously, it was suggested that these exercise-related improvements may be due to normalization of myosin isoenzyme distribution in the diabetic heart [2,9]. However, cardiac MHC isoform mRNA and protein composition was not changed following long-term treadmill training (8–10 weeks) in diabetic and non-diabetic rodents [13,15]. In contrast, our acute results revealed a different pattern. We observed that myocardial MHC-α mRNA and MHC-β mRNA expressions were acutely normalized after a single exercise session, but notably, only in the diabetic and not the normal rats. These findings suggest that the acute cardiac MHC isoform mRNA expression in response to exercise is consistent with the long-term response in the healthy heart but not in the diabetic heart. It has been suggested that the predominance of the cardiac MHC-α isoform may influence its expression in response to exercise. Specifically, when MHC-α is predominant, exercise may induce minimal or no shift toward the MHC-α isoform [28]. Our findings appear to align with this speculation, as a single exercise session did not induce any increase in MHC-α isoform mRNA expression compared to no exercise in the normal heart, which is predominantly composed of MHC-α. This implies that, over the longer term, it is less likely for exercise to induce an increase in the MHC-α isoform composition in the normal heart, in accordance with the previous suggestion [28]. In contrast, the diabetic heart, where MHC-β predominates, exhibited a reduction in MHC-β and an increase in MHC-α mRNA isoform following a single session of exercise. These differences underscore the role of basal cardiac MHC isoform composition in determining the responsiveness to exercise. 

The observed variations in myocardial MHC mRNA responses in diabetic status may be due to the different types and intensities of exercise in acute versus long-term exercise training protocols. In our study, the running protocol consisted of a single session of 60 min at 50 m/min and 0% grade, while the previous protocol involved running 60 min per day at 27 m/min and 10% grade [13]. It is difficult to compare the two protocols in terms of maximal oxygen uptake percentage due to these notable differences in speed and grade. Nonetheless, both protocols involved different intensities, which might have resulted in inconsistent myosin heavy-chain isoform responses in the diabetic heart. In support of this potential distinct impact of exercise type and intensity, the function of diabetic hearts was improved by long-term swimming exercise (90 min per day) in association with partially reversed myocardial MHC-α isoform content in diabetic rats [29]. Unfortunately, it is unknown if this type of swimming exercise can acutely change the myosin heavy-chain isoform mRNA in a similar pattern after the long-term exercise regimen. Nonetheless, whether the acute and long-term myocardial MHC isoform mRNA and protein responses to exercise in diabetes are consistent has not yet been determined. In future research, investigating these responses in diabetes should be performed through maintaining similarity in exercise type and intensity across both acute and long-term exercise regimens while also exploring these responses across a range of intensities.

Nevertheless, the acute response of MHC isoform mRNA expression to a single exercise session in the diabetic heart may be implicated in the cardiac adaptations to exercise training through different mechanisms other than chronically changing the myocardial MHC isoform composition. For example, MHC mRNA was shown to influence the encoding of a network of microRNAs, including microRNA-208, which are closely associated with heart health and disease [16,17]. MicroRNAs have been associated with different heart conditions such as heart failure, cardiac hypertrophy, and myocardial infarction, and are involved in the heart’s response to stress and thyroid hormone [30]. Consequently, acute changes in MHC isoform mRNA expression following a single exercise session may contribute to improved diabetic heart contractility observed with long-term training by triggering mechanisms that may ameliorate heart stress-related responses. Further investigations are required to elucidate the immediate and long-term implications of MHC isoform mRNA changes in cardiac adaptations. 

One of the current noteworthy findings is that TR- α1 mRNA was elevated in diabetic rats compared to non-diabetic rats and that a single exercise session completely normalized its expression. This contrasts with previous findings that showed TRs, α1 and β1, were not changed at the levels of mRNA and protein in the left ventricle of diabetic rats despite the shift in MHC isoform distribution to MHC-β [8]. Haddad et al. quantified TRs mRNA expression at 3 and 6 weeks post-STZ injection using Northern analysis. The different methodologies employed for measuring mRNA expression in both their study and in ours might be the reason for these conflicting findings. TR-α1 is the dominant isoform in cardiac cells that interacts with the thyroid hormone axis to increase the expression of MHC-α and reduce the expression of MHC-β [31,32]. Considering the shift in cardiac MHC isoform composition towards MHC-β dominance in diabetes, we initially hypothesized that TR-α1 would decrease in the diabetic heart. Contrary to our expectations, TR-α1 was largely increased in the diabetic rats. The active form of thyroid hormone, T3, has been reported to decrease in the diabetic state [33], without a concurrent increase in TR-α1 binding affinity [8]. Accordingly, the increased cardiac TR-α1 mRNA expression with STZ-induced diabetes could be a compensatory mechanism to counteract the reduced T3 levels, a theory that necessitates further investigations and validation. 

Interestingly, cardiac TR-α1 mRNA was significantly reduced after a single session of exercise concurrent with cardiac MHC-α and MHC-β mRNA normalization. As discussed earlier, it is challenging to interpret these findings. T3 has been reported to immediately increase after a single session of exercise at different intensities in healthy humans and rats [34,35]. There are lack of data regarding the acute exercise effect on T3 in individuals with diabetic status. If we extrapolate these results to diabetes, the acute increase in T3 after exercise, which is already absent or at low levels in diabetics, may stimulate its receptors TR-α1 and associated genes expression effects, including MHC-α mRNA expression and MHC-β mRNA suppression. Thus, it is plausible that the suppression and/or degradation of TR-α1 mRNA occurred through specific negative feedback mechanisms, which may have been triggered by TR-α1 reactivation after being inactive. Unfortunately, we lack sufficient data to confirm these speculations, highlighting potential aims of future studies in the field. 

One of the limitations of this study is that we did not measure T3 level and TR-α1 protein expression. Had we possessed these data, we could have unraveled, at least partly, whether the T3 axis and TR-α1 receptors were involved in the acute normalization of MHC isoforms in diabetic hearts. Furthermore, missing these data prevented us from determining whether the increased TR-α1 mRNA was associated with increased TR-α1 receptor levels or if the mRNA degradation occurred before translation. Another limitation of this study is that we were unable to perform functional and structural tests to validate cardiomyopathy due to a lack of equipment. Instead, we followed previously well-established procedures (as detailed in the Methodology Section) that successfully led to the development of cardiomyopathy in a similar rat strain [23,24,25].

## 5. Conclusions

In summary, our study demonstrates that acute modulation of MHC and TR isoform mRNA expression can occur after a single exercise session. This acute effect is prominent primarily in the diabetic heart, whereas no changes occur in the normal heart. Nevertheless, it remains uncertain whether these acute responses in mRNA expression induce chronic changes in mRNA composition. Moreover, the question of whether inhibitory mechanisms prevent protein translation during long-term exercise training in diabetic hearts remains unknown. Additionally, we speculate on the possibility that these acute changes in the MHC isoform mRNA expression may trigger other mechanisms, such as microRNA effects, that contribute to improved diabetic heart structure and function after exercise training. These findings raise crucial questions for future research.

## Figures and Tables

**Table 1:** Primers sequences utilized for RT-PCR.

Gene	Forward Primer	Reverse Primer
*GAPDH*	ATGGTGAAGGTCGGTGTG	GAACTTGCCGTGGGTAGA
*MHC-α*	GGGACCGTAGCAAGAAGGACAATC	AACTTCCCAAAGCGGGAGGAGT
*MHC-β*	TGGAGCTGATGCACCTGTAGAC	GATGATGCAGCGTACAAAGTGAGG
*TR-α1*	GCCGCTTCCTCCACATGAAAGTC	CCCAGCTTTGTCCCTTCTCTCCA
*TR-β*	CCGGAAGGTGGCAAGGTTGATCT	GGTCTTCACAGGGCAGCTCACAAA

GAPDH, Glyceraldehyde-3-Phosphate Dehydrogenase; MHC-α, myosin heavy-chain α isoform; MHC-β, myosin heavy-chain β isoform; TR-α1, thyroid receptor α1 isoform; TR-β, thyroid receptor β isoform.

**Table 2:** Body characteristics.

Group	Initial BW (g)	Final BW (g)	HW	HW/BW
CS	231.8 ± 25.2	267.5 ± 22.0	0.86 ± 0.1 #	0.0032 ± 0.0004 $
CEX	227.2 ± 13.9	289.4 ± 16.5 *#	1.03 ± 0.1 ‡#$	0.0036 ± 0.0003 $
DIS	203.3 ± 24.8	173.3 ± 26.2 ‡	0.63 ± 0.15 ‡	0.0036 ± 0.0006 $
DIEX	205.0 ± 25.4	216.1 ± 89.9	0.88 ± 0.15 #	0.0043 ± 0.001

Values are expressed as mean ± SD. CS—non-diabetic control; CEX—non-diabetic exercise; DIS—sedentary diabetic; DIEX—diabetic exercise; BW—body weight; HW—heart weight; HW/BW—heart weight to body weight ratio. * Significant difference with initial body weight at *p* < 0.05. ‡ Significant difference with CS at *p* < 0.05. # Significant difference with DIS at *p* < 0.05. $ significant difference with DIEX.

## Data Availability

The data are available upon specific and reasonable request through direct contact with the corresponding author.
